# In-Situ Synthesis of Heterostructured Carbon-Coated Co/MnO Nanowire Arrays for High-Performance Anodes in Asymmetric Supercapacitors

**DOI:** 10.3390/molecules25143218

**Published:** 2020-07-15

**Authors:** Guoqing Chen, Xuming Zhang, Yuanhang Ma, Hao Song, Chaoran Pi, Yang Zheng, Biao Gao, Jijiang Fu, Paul K. Chu

**Affiliations:** 1 The State Key Laboratory of Refractories and Metallurgy, Institute of Advanced Materials and Nanotechnology, Wuhan University of Science and Technology, Wuhan 430081, China; chenguoqing1994@sina.com (G.C.); yuanhang_ma@163.com (Y.M.); songhao201809@wust.edu.cn (H.S.); chaoran_pi@wust.edu.cn (C.P.); gaobiao@wust.edu.cn (B.G.); fujijiang@wust.edu.cn (J.F.); 2 Department of Physics, City University of Hong Kong, Tat Chee Avenue, Kowloon, Hong Kong, China; 3 Department of Materials Science & Engineering, City University of Hong Kong, Tat Chee Avenue, Kowloon, Hong Kong, China; 4 Department of Biomedical Engineering, City University of Hong Kong, Tat Chee Avenue, Kowloon, Hong Kong, China

**Keywords:** pseudocapacitors, conductivity, manganese oxide, cobalt, core-shell structure

## Abstract

Structural design is often investigated to decrease the electron transfer depletion in/on the pseudocapacitive electrode for excellent capacitance performance. However, a simple way to improve the internal and external electron transfer efficiency is still challenging. In this work, we prepared a novel structure composed of cobalt (Co) nanoparticles (NPs) embedded MnO nanowires (NWs) with an N-doped carbon (NC) coating on carbon cloth (CC) by in situ thermal treatment of polydopamine (PDA) coated MnCo_2_O_4.5_ NWs in an inert atmosphere. The PDA coating was carbonized into the NC shell and simultaneously reduced the MnCo_2_O_4.5_ to Co NPs and MnO NWs, which greatly improve the surface and internal electron transfer ability on/in MnO boding well supercapacitive properties. The hybrid electrode shows a high specific capacitance of 747 F g^−1^ at 1 A g^−1^ and good cycling stability with 93% capacitance retention after 5,000 cycles at 10 A g^−1^. By coupling with vanadium nitride with an N-doped carbon coating (VN@NC) negative electrode, the asymmetric supercapacitor delivers a high energy density of 48.15 Wh kg^−1^ for a power density of 0.96 kW kg^−1^ as well as outstanding cycling performance with 82% retention after 2000 cycles at 10 A g^−1^. The electrode design and synthesis suggests large potential in the production of high-performance energy storage devices.

## 1. Introduction

Growing concerns about environmental pollution arising from consumption of fossil fuels have spurred the development of green and sustainable energy and efficient energy storage devices for portable electronics and electric vehicles are in high demand [1,2]. Supercapacitors (SCs) can meet the requirements for electrochemical energy storage due to the rapid charging capability, high specific power capacity, as well as superior lifetime [3,4,5,6]. According to the charge storage mechanisms, SCs can be classified as electrical double-layer capacitors (EDLCs) and faradic pseudocapacitors. Faradic pseudocapacitors incorporating transition metal oxides (TMOs) such as MnO_2_, Co_3_O_4_, NiO, and RuO_2_ or conducting polymers like polypyrrole, polyaniline, and polythiophene have been widely studied because of high specific capacitance as a result of multiple redox reactions and multi-electron transfer [5,7,8,9,10,11,12,13].

Among the various types of TMOs, manganese oxide (MnO_x_, 1 ≤ x ≤ 2) is attractive on account of the low cost, chemical stability, large natural abundance, and high theoretical capacitance of about 1370 F g^−1^ [14,15,16,17,18]. However, the poor electrical conductivity (10^−5^–10^−6^ S cm^−1^) restricts rapid charge transfer during charging/discharging resulting in inferior rate capacity and limited cyclic stability [19,20]. Hence, more efficient structures must be designed to make it commercial viable [21,22,23,24]. Such as core-shell MnO@N-rich carbon nanosheets as the positive supercapacitor electrode with a capacitance of 570 F g^−1^ at 2 A g^−1^ as well as capacitance retention of 99% for 6000 cycles [18], hierarchical porous and hollow core-shell MnO_2_@C microspheres with a specific capacitance of 280 F g^−1^ at a current density of 0.1 A g^−1^ [25], amorphous MnO_2_ decorated TiC/C core/shell nanofiber electrode with a high specific discharge capacity of 645 F g^−1^ at 1 A g^−1^ together with 99% capacity retention after 5000 charging/discharging cycles [26]. However, most TMOs based electrodes have unsatisfactory capacitive properties as a result of the limited utilization efficiency of the large size or thick TMOs because of the high internal resistance limited the internal electron transfer to outside [27]. Metal NPs incorporated into TMOs have been reported to enhance capacitive performance by reducing the electron transfer depletion, such as Ag-Co_3_O_4_ [28], Au-MnO_2_ [29], and Au-NiO [30]. Noble metals with high stability are most suitable, but it was restricted by the high cost and complex preparation process. Other non-noble metals are promising alternatives, but the facile fabrication process is still challenging.

**Scheme 1:**
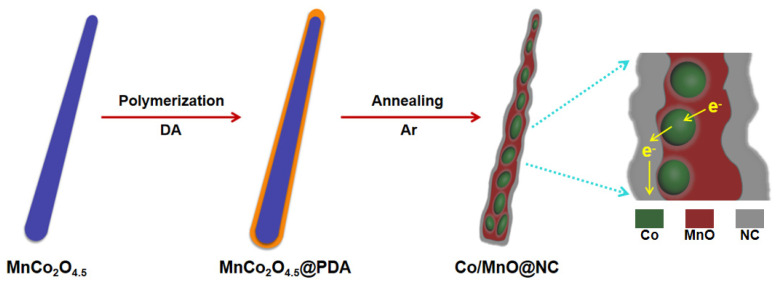
Illustration of the formation process of the Co/MnO@NC core–shell nanowires (NWs).

Herein, a facile strategy to produce a core-shell structure composed of Co NPs embedded MnO nanowires with an N-doped carbon coating on carbon cloth (Co/MnO@NC NWs) by one-step thermal treatment of PDA-coated MnCo_2_O_4.5_ (MnCo_2_O_4.5_@PDA) nanowire arrays in Ar is described. The NWs on CC with strong adhesion obviates the need for an organic binder and provides high electron transport efficiency to the current collector [31,32,33]. As shown in Scheme 1, the coated PDA is carbonized into the NC shell and the MnCo_2_O_4.5_ core is reduced simultaneously to Co NPs and MnO. The Co NPs are uniformly dispersed in the MnO matrix. Consequently, the Co/MnO@NC NWs show a high specific capacitance of 747 F g^−1^ at 1 A g^−1^ and good cycling stability with 93% capacitance retention after 5000 cycles at 10 A g^−1^. The asymmetric supercapacitor constructed with Co/MnO@NC as the positive electrode and VN@NC as the negative electrode shows a high energy density of 48.15 Wh kg^−1^ and power density of 0.96 kW kg^−1^. It remains at 27.9 Wh kg^−1^ for a higher power density of 18.95 kW kg^−1^ and the promising electrode materials are suitable for high-performance electrochemical energy storage devices.

## 2. Experimental Details

### 2.1. Synthesis of MnCo_2_O_4.5_NWs

All the reagents (analytical grade) were purchased from Aladin Ltd. (Shanghai, China) and used without purification. Briefly, 4 mM CoCl_2_ × 6H_2_O, 2 mM MnCl_2_ × 4H_2_O, 24 mM urea, and 10 mM NH4F were dissolved in deionized water (70 mL) under mild magnetic stirring at room temperature for 30 min to obtain a pink transparent solution that was subsequently transferred to a Teflon-lined stainless steel autoclave (200 mL). A piece of CC (2 cm × 6 cm) was ultrasonically cleaned with deionized water, acetone, and alcohol and put into the autoclave and maintained at 120 °C for 5 h. After the autoclave cooled to room temperature, the precursor NWs was produced on CC. The pristine product (MnCo_2_O_4.5_ NWs) with a mass loading of about 3 mg cm^−2^ was obtained by calcination in air at 350 °C for 2 h.

### 2.2. Preparation of Co/MnO@NC NWs

The as-prepared MnCo_2_O_4.5_ NWs on CC were immersed in a mixture containing H_2_O (100 mL), Tris-HCl buffer (121 mg), and dopamine (DA 400 mg) and magnetically stirred for 8 h. The sample (MnCo_2_O_4.5_@PDA NWs) was taken out, washed several times to remove the remaining DA, and dried at 60 °C. The MnCo_2_O_4.5_@PDA NWs were then sintered at 500 °C, 600 °C, and 700 °C for 3 h in Ar (samples labeled as S-500, S-600, and S-700) with the mass loading reduced to about 2 mg cm^−2^. For comparison, MnCo_2_O_4.5_ NWs without the PDA coating were treated at 600 °C (MnCo_2_O_4.5_-Ar(600)).

### 2.3. Preparation of VN@NC NWs 

The VN@NC NWs were prepared by a solvothermal technique described previously [34]. 2 mM V_2_O_5_ powder and 6 mM H_2_C_2_O_4_ powder were dissolved in 12 mL of deionized water under rigorous stirring at 75 °C for 1 h until a clear dark blue solution was formed. 3 mL of 30% H_2_O_2_ were added and stirred for about 30 min to obtain a brown solution. After adding 30 mL of ethanol, the solution was transferred to a Teflon-lined stainless autoclave (100 mL) with a piece of carbon cloth (2 cm × 6 cm) and heated to 180 °C for 2 h. After the hydrothermal treatment, the VO_x_ NWs on CC were rinsed with distilled water and ethanol several times and coated with PDA by the same method. Finally, the VN@NC NWs were produced by annealing at 600 °C in NH_3_ for 2 h.

### 2.4. Materials Characterization

The morphology and crystal structure of the samples were characterized by field-emission scanning electron microscopy (FE-SEM, FEI Nova 450 Nano), transmission electron microscopy (TEM, JEOL 2010), X-ray diffraction (XRD, Philipa X’Pert Pro), and X-ray photoelectron spectroscopy (XPS, PHI-5000 V). Raman scattering was conducted using a 532 nm argon laser as the excitation source on the WITec-CRM200 Raman system (WITec, Ulm, Germany).

### 2.5. Electrochemical Assessment

The electrochemical measurements were performed using a three-electrode configuration on an electrochemical workstation (CHI760E) in 6 M KOH. The materials with an exposed area of 1 cm × 1 cm, platinum (Pt) plate, and saturated calomel electrode (SCE) served as the working electrode, counter electrode, and reference electrode, respectively. Cyclic voltammetry (CV) was conducted from 0 to 0.5 V at different scanning rates (5–50 mV s^−1^) and galvanostatic charge-discharge (GCD) measurements were carried out at current densities from 1 to 20 A g^−1^. Electrochemical impedance spectroscopy (EIS) was performed in a frequency range between 10 mHz and 100 kHz with an AC perturbation of 5 mV. All of the specific capacitances were calculated from the galvanostatic charge/discharge (GCD) curves by the following equation:

C_s_ = I × ∆t/(m_1_ × ∆V) (1)

where C_s_ (F g^−1^) stands for the specific capacitance of electrode, I (A) represents the discharging current, Δt (s) is the discharge time, ΔV is the voltage range, and m_1_ (g) is the mass of active materials on CC.

### 2.6. Preparation of Asymmetric Supercapacitor

An asymmetric device was prepared with the Co/MnO@NC NWs being the positive electrode, VN@NC NWs as the negative electrode, and a separator in a button cell (Standard CR2032-type). The electrochemical tests were performed in different potential ranges in a two-electrode electrochemical cell. The specific capacitance of the device (C) was calculated from the galvanostatic charge-discharge (GCD) curves by the following equation:

C = I × ∆t/((m^+^ + m^−^) × ∆V) (2)

where I is the discharge current, ΔV is the voltage range, Δt is the discharge time, and (m^+^ + m^−^) is the total mass of the active materials on both electrodes matched by the following charge balancing equation:

m^+^ × C^+^ × ΔV^+^ = m^−^ × C^−^ × ΔV^−^ (3)

where m^+^ and m^−^ represent the active mass loadings of the positive and negative materials, C^+^ and C^−^ are the positive and negative gravimetric specific capacitances, and ΔV^+^ and ΔV^−^ stand for the operating voltage window of the positive electrode and negative electrodes, respectively. The gravimetric energy and power densities of the asymmetric supercapacitor were calculated as follows:

E = C × ΔV^2^/7.2 (4)

P = 3.6 E/Δt(5)

where E (Wh kg^−1^) is the energy density, P (kW kg^−1^) represents the power density, ∆V is the voltage window range, and ∆t (s) stands for the discharging time.

## 3. Results and Discussion

**Figure 1:**
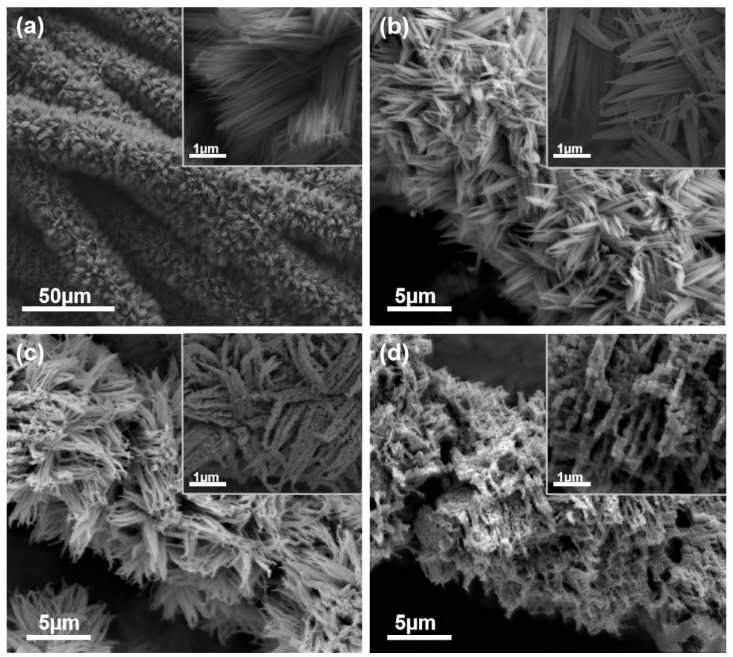
SEM images: (**a**) Pristine MnCo_2_O_4.5_, (**b**) S-500, (**c**) S-600, and (**d**) S-700.

Figure 1a presents the morphology of pristine MnCo_2_O_4.5_ NWs which are uniformly distributed on the carbon cloth. The length is about 5 μm and the surface is smooth. After sintering the PDA-coated MnCo_2_O_4.5_ NWs at 500 °C under Ar, the NWs (S-500) become thicker but the length is similar because of the PDA coating (Figure 1b). When the sintering temperature is increased to 600 °C (Figure 1c), the surface of S-600 becomes relatively rough, and the structure collapses at 700 °C (Figure 1d). Without the PDA coating, the bare MnCo_2_O_4.5_ NWs thermally treated at 600 °C in Ar retain the original morphology as the pristine product (Figure S1) contrary to the PDA-coated sample.

**Figure 2:**
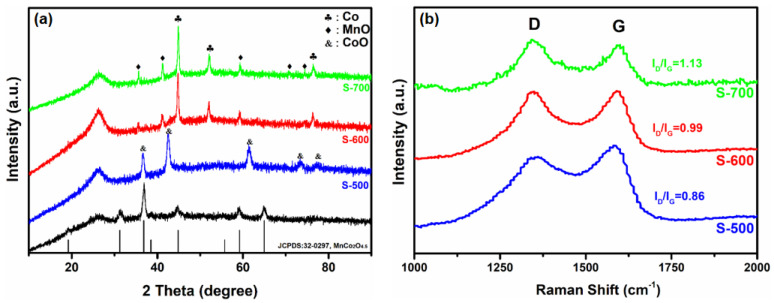
(**a**) XRD patterns of the pristine MnCo_2_O_4.5_, S-500, S-600, and S-700; (**b**) Raman scattering spectra of S-500, S-600, and S-700.

The X-ray diffraction (XRD) pattern of the pristine product (black line) in Figure 2a shows diffraction peaks at 19°, 31.2°, 36.8°, 38.5°, 44.8°, 55.7°, 59.5°, and 65.3° corresponding to the (111), (220), (311), (222), (400), (422), (511), and (440) planes of spinel MnCo_2_O_4.5_ (JCPDS card 32-0297) [35,36,37] in addition to the characteristic peak at 26.5° associated with the (002) plane of CC. After the PDA-coated NWs treated at 500 °C (S-500, blue line), the diffraction peak of spinel MnCo_2_O_4.5_ disappears and new broad peaks arising from CoO (JCPDS card 78-0431) and overlapping peaks from the characteristic peaks of MnO (JCPDS card 75-0625) appear indicating that the MnCo_2_O_4.5_ NWs have been converted to a mixture of MnO/CoO NWs consistent with previous reports [38]. The crystal phase of CoO vanishes at 600 °C (S-600, red line) and is replaced by peaks at 44.3°, 51.5°, and 75.9° corresponding to the (111), (200), and (220) crystal planes of metallic Co (JCPDS card 89-7093) and sharp diffraction peaks of MnO [38,39,40]. The strong spinel crystal phase and fractional CoO phase can be observed from MnCo_2_O_4.5_-Ar(600) without the PDA coating (Figure S2) suggesting that spinel MnCo_2_O_4.5_ is not stable at the high temperature. XRD indicates that the PDA coating annealing at high temperature have strong reduction effects. At a high annealing temperature (S-700, green line), there are no new diffraction peaks but the structure collapses totally as shown by the SEM images. The Raman scattering spectra of S-500, S-600, and S-700 are presented in Figure 2b. All the samples exhibit two strong Raman peaks at 1350 and 1590 cm^−1^ corresponding to the D and G bands, respectively, suggesting conversion of the organic PDA coating to carbon. The I_D_/I_G_ ratios of S-500, S-600, and S-700 are 0.86, 0.99, and 1.13, respectively. The I_D_/I_G_ ratio increases with sintering temperature due to the interactions between graphite carbon and metal oxide. The porous microstructure of the NWs and graphite are expected to deliver good electrochemical performance [38,41].

**Figure 3:**
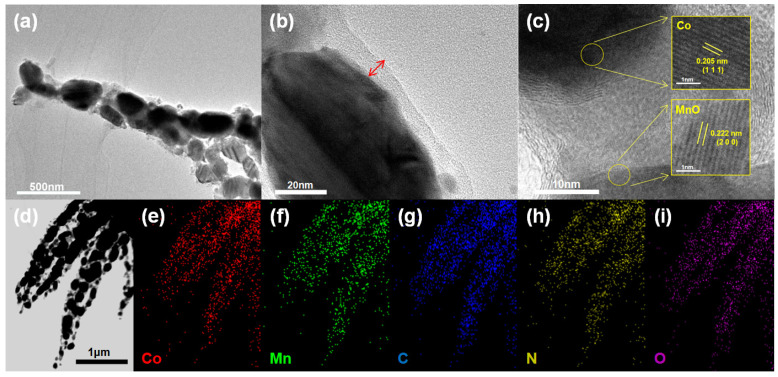
(**a**,**b**) TEM images of S-600, (**c**) HR-TEM image, and (**d**–**i**) Elemental maps of S-600.

The microstructure and chemical composition of Co/MnO@NC (S-600) are characterized by TEM and elemental mapping. Figure 3a shows the wire-like structure consisting of large particles several hundred nanometers in size and lots of gap between the nanoparticles. A thin carbon coating about 7 nm thick is present on the particle as shown in Figure 3b. The HR-TEM image in Figure 3c reveals lattice fringes of 0.205 nm and 0.222 nm corresponding to the (111) plane of metallic Co and (200) plane of MnO, respectively. Figure 3d–i show many black grains derived from high-density metallic Co nanoparticles uniformly dispersed in the NWs. The elemental maps obtained by EDS reveals that Co, Mn, C, N, and O are uniformly distributed in the nanowires and N-doped carbon encapsulates the Co/MnO NWs forming the porous Co/MnO@NC NWs.

**Figure 4:**
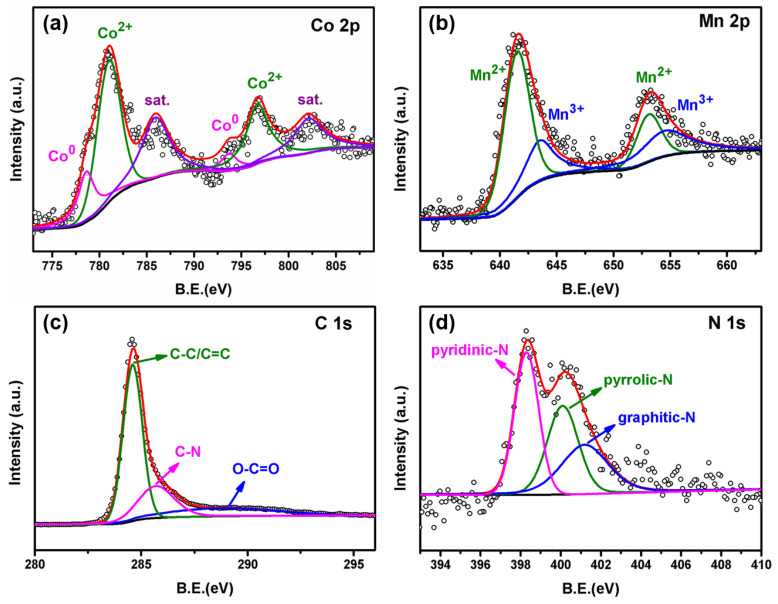
XPS spectra of S-600: (**a**) Co 2p, (**b**) Mn 2p, (**c**) C 1s, and (**d**) N 1s.

X-ray photoelectron spectroscopy (XPS) is conducted to determine the composition and chemical states of the S-600 NWs. Figure S3 exhibits the XPS spectrum revealing the presence of Co, Mn, C, N, and O. The high-resolution Co 2p spectrum in Figure 4a can be deconvoluted into three pair of peaks. The two doublets at 778.5/793.7 eV and 780.5/796.7 eV can be ascribed to the Co^0^ and Co^2+^ in addition of the satellite peak of 785.9/802.1 eV [42,43]. The Mn 2p spectrum in Figure 4b shows two prominent peaks at 641.5 eV and 653.4 eV due to Mn^2+^ in the oxide in addition to the high oxide state of Mn^3+^ (643.5/654.5 eV) [44,45,46]. Figure 4c shows the high-resolution C 1s spectrum consisting of a dominant peak at 284.7 eV and two minor peaks at 285.7 eV and 288.8 eV related to C-C/C=C, C-N, and O-C=O, respectively [47]. The C-N bond confirms the formation of the N-doped carbon coating by carbonization of PDA. Figure 4d shows the N 1s spectrum and the three peaks at 398.9, 399.8, and 401.6 eV are associated with pyridinic-N, pyrrolic-N, and graphitic-N, respectively [47,48]. However, MnCo_2_O_4.5_-Ar(600) without the PDA coating shows no N 1s and Co^0^ (Figure S4) suggesting that MnCo_2_O_4.5_ without the carbon coating cannot be converted into the Co/MnO composite as consistent with XRD.

**Figure 5:**
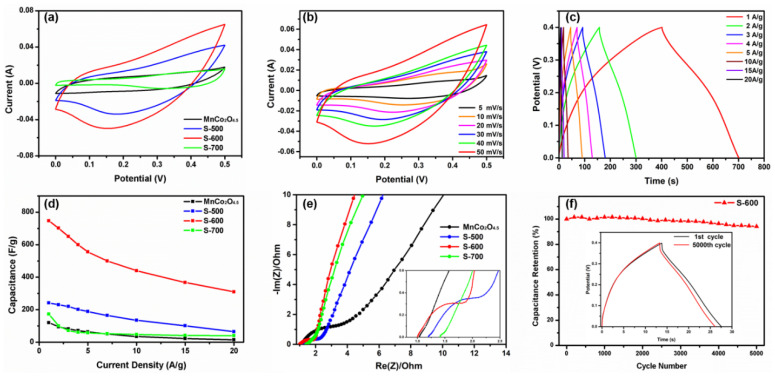
(**a**) Cyclic voltammetry (CV) curves of the pristine MnCo_2_O_4.5_, S-500, S-600, and S-700 at a scanning rate of 50 mV s^−1^; (**b**) CV curves of S-600 at different scanning rates; (**c**) galvanostatic charge–discharge (GCD) curves of S-600; (**d**) Specific capacitances; (**e**) Nyquist plots; (**f**) Cycling performance of S-600 at 10 A g^−1^.

The electrochemical measurements are carried out on a three-electrode system in 6 M KOH. Figure 5a shows the cyclic voltammetry (CV) curves of the pristine MnCo_2_O_4.5_, S-500, S-600, and S-700 electrodes at a scanning rate of 50 mV s^−1^. The largest CV area is observed from the S-600 electrode indicating the largest supercapacitive capacity. The CV curves of S-600 acquired at different scanning rates in the voltage range of 0–0.5 V (vs. SCE) are displayed in Figure 5b. The shape of the CV curves indicates that the capacity derives mainly from MnO and a part of CoO on the surface of the Co NPs. Figure 5c shows the galvanostatic charge-discharge (GCD) curves of the S-600 electrode in the current density range between 1 and 20 A g^−1^. The symmetrical charge-discharge curves reveal the reversible faradaic redox reaction. The specific capacitances of the S-600 electrode are 747, 702, 650, 600, 556, 440, 367, and 310 F g^−1^ at current densities of 1, 2, 3, 4, 5, 10, 15, and 20 A g^−1^ (Figure 5d), respectively, which are much higher than those of the pristine MnCo_2_O_4.5_ (120 F g^−1^ at 1 A g^−1^) and other electrodes. The IR drop in the GCD curves of the pristine MnCo_2_O_4.5_ and S-500 electrodes indicate the high intrinsic resistance (Figure S5). Figure 5e shows the electrochemical impedance spectroscopy (EIS) results. All the Nyquist plots show the typical electrochemical capacitive behavior with a straight line in the low frequency region and a semicircle in the high frequency region indicating the ion diffusion resistance and charge transfer resistance between the electrode interface and electrolyte, respectively. The slope of the straight line of S-600 in the low frequency region is steeper than that of the other electrodes revealing lower ion diffusion resistance because of the porous structure. And the intercept of X-axis in the high frequency region for S-600 is smaller than other electrodes, showing that S-600 has smallest contact resistance with electrolyte (Figure 5e). It is noted that the S-700 shows high contact resistance, but the physical resistance is smallest, which could attribute to the hydrophobic graphite carbon. However, the high electrochemical active surface area was dramatically decreased due to the collapse structure at high annealing temperature which worsen the electrochemical performance (Figure S6). Moreover, the electrochemical performance of MnCo_2_O_4.5_-Ar(600) without carbon coating is much less than S-600 (Figure S7). Figure 5f presents the long-term cycling stability of S-600 for a GCD current density of 10 A g^−1^ and 93% of the specific capacitance is retained after 5000 cycles. The morphology of the porous NWs does not change (Figure S8) confirming the structural robustness. The excellent electrochemical properties of S-600 can be attributed to the novel structure. Firstly, the carbonized NC shell improves the structural stability and surface conductivity of the NWs to achieve excellent cycling stability. Secondly, the in situ formed Co NPs dispersed in the MnO matrix enhances the efficiency of electron transfer from inside MnO to outside boosting high specific capacity and high rate capability. Thirdly, the porous NWs with high electrochemical active surface area allow fast diffusion of the electrolyte into the inner region of the electrode further improving the capacitive performance.

**Figure 6:**
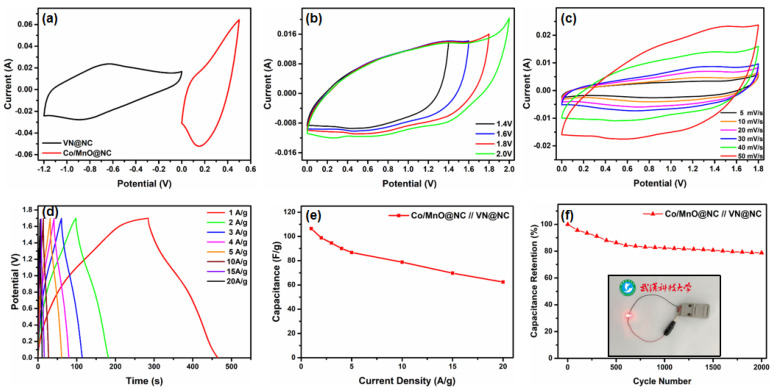
Electrochemical performance of the asymmetric supercapacitor (ASC) device assembled with Co/MnO@NC//VN@NC: (**a**) CV curves of the two electrodes at a scanning rate of 50 mV s^−1^; (**b**) CV curves in different potential ranges; (**c**) CV curves at different scanning rates; (**d**) GCD curves; (**e**) specific capacitances; (**f**) cycling performance at 10 A g^−1^.

To assess the commercial potential, an asymmetric supercapacitor (ASC) with two electrodes is assembled as a button cell with S-600 as the positive electrode and VN@NC as the negative electrode (Co/MnO@NC//VN@NC). VN has a wide potential window and excellent capacitive behavior and should improve the energy density of the ASC. The VN@NC NWs are prepared on CC by a hydrothermal reaction, PDA coating, and nitridation and more details are available from our previous paper [34]. The VN@NC NWs are distributed on the carbon cloth uniformly (Figure S9) and have similar specific capacitance and mass loading as Co/MnO@NC (Figure S10). Figure 6a shows the CV curves of the two electrodes at a scanning rate of 50 mV s^−1^ and stable and matched capacitance are obtained in the voltage windows between 0 and 0.5 V for S-600 and −1.2 and 0 V for VN@NC. The CV curves of the ASC in the different potential window ranges of 0–1.4 V, 0–1.6 V, 0–1.8 V, and 0–2.0 V are displayed in Figure 6b. The rectangular shape indicates an excellent capacitive behavior and reversibility. Figure 6c displays the CV curves of the ASC at different scanning rates in the voltage range of 0–1.8 V and the rectangular shape implies fast charging/discharging. The GCD plots of the ASC at different current densities in Figure 6d show nearly symmetrical charging/discharging curves indicative of the good reversible Faradaic redox reaction. The total specific capacitance of the ASC reaches a maximum of 107 F g^−1^ at 1 A g^−1^ and remains at 62 F g^−1^ at 20 A g^−1^ (Figure 6e). The gravimetric energy density of the ASC is about 48.15 Wh kg^−1^ for a power density of 0.96 kW kg^−1^ and remains at 27.9 Wh kg^−1^ for a power density of 18.95 kW kg^−1^. The properties are comparable with or better than those of previously reported materials such as MnCo_2_O_4.5_//AC (40.5 Wh kg^−1^ at 376 W kg^−1^) [36], MnO_2_@CNT//MoO_3_@CNT (27.8 Wh kg^−1^ at 524 W kg^−1^) [49], Ni(OH)_2_-MnO_2_-RGO//RGO (54.0 Wh kg^−1^ at 392 W kg^−1^) [50], Fe_2_O_3_//MnO_2_ (22 Wh kg^−1^ at 106 W kg^−1^) [51], and other supercapacitive devices [18,52,53]. The cycling stability of the ASC is evaluated by GCD at a current density of 10 A g^−1^ as shown in Figure 6f. The ASC exhibits remarkable cycling stability and capacitance retention of about 84% after 500 cycles. After that, the capacitance remains stable at 82% of the initial capacitance after 2000 cycles. The inset picture shows that a red light-emitting diode (LED) with a threshold voltage of 1.8 V can be powered by one button cell.

## 4. Conclusions

Co/MnO@NC core–shell NWs are prepared by a thermal treatment of PDA-coated MnCo_2_O_4.5_ NWs in Ar. The coated PDA is carbonized into the NC shell and the MnCo_2_O_4.5_ core is converted to Co NPs and MnO with a uniform distribution. The surface and internal conductivity of the active MnO is improved by the conductive NC shell and embedded Co NPs and the diffusion efficiency of the electrolyte is enhanced by the porous structure. The Co/MnO@NC NWs has a remarkable capacitance of 747 F g^−1^ at a current density of 1 A g^−1^ and good long-term cycling stability with 93% retention after 5000 cycles at a high current density of 10 A g^−1^. In addition, the assembled asymmetric supercapacitor (Co/MnO@NC//VN@NC) shows a high capacitance of 107 F g^−1^ at 1 A g^−1^ and gravimetric energy densities of 48.15 Wh kg^−1^ and 27.9 Wh kg^−1^ for power densities of 0.96 kW kg^−1^ and 18.95 kW kg^−1^, respectively. The cycling stability is also outstanding with 82% retention after 2000 cycles. The Co/MnO@NC NWs with excellent properties have large potential in high-performance electrochemical energy storage devices.

## Supplementary Materials

The following are available online, Figure S1: SEM image of MnCo_2_O_4.5_-Ar(600), Figure S2: XRD patterns of the pristine MnCo_2_O_4.5_ and MnCo_2_O_4.5_-Ar(600), Figure S3: Survey scanning XPS spectra of the S-600, Figure S4: (**a**) Survey scanning XPS spectra of the MnCo_2_O_4.5_-Ar(600), (**b**) and (**c**) Corresponding high resolution Co 2p and Mn 2p, Figure S5: CV and GCD curves of (**a**,**b**) pristine MnCo_2_O_4.5_ NWs, (**c**,**d**) S-500 and (**e**,**f**) S-700, Figure S6: Current densities obtained from CV curves as a function of scanning rates, the corresponding slope being the C_dl_ values, Figure S7: Electrochemical performance: (**a**) CV curves of MnCo_2_O_4.5_, MnCo_2_O_4.5_-Ar(600) and S-600 at a scanning rate of 50 mV s^−1^, (**b**) GCD curves at 1 A g^−1^, (**c**) Specific capacitances, (**d**) Nyquist plots, Figure S8: SEM image of S-600 NWs after 5000 cycles, Figure S9: (**a**) SEM image of VN@NC, (**b**) Corresponding XRD pattern, Figure S10: Electrochemical performance of VN@NC NWs electrode, (**a**) CV curves, (**b**) GCD curves, (**c**) Corresponding specific capacitances as function of discharging density.
