# Supplementary material for: In-Situ Synthesis of Heterostructured Carbon-Coated Co/MnO Nanowire Arrays for High-Performance Anodes in Asymmetric Supercapacitors
Source: Molecules. 2020 Jul 15;25(14):3218. doi: 10.3390/molecules25143218 (PMC7397025; doi:10.3390/molecules25143218)
Supplement: Supplementary file 1 [file molecules-25-03218-s001.pdf]

Supporting Information

In-Situ Synthesis of Heterostructured Carbon-Coated Co/MnO Nanowire Arrays for High-Performance Anodes in Asymmetric Supercapacitors

Guoqing Chen,[a] Xuming Zhang,[a*] Yuanhang Ma,[a] Hao Song,[a,b] Chaoran Pi,[a] Yang Zheng,[a*] Biao Gao,[a,b] Jijiang Fu,[a] and Paul K Chu[b*]

[a] The State Key Laboratory of Refractories and Metallurgy, Institute of Advanced Materials and Nanotechnology, Wuhan University of Science and Technology, Wuhan 430081, China.

[b] Department of Physics, Department of Materials Science & Engineering, and Department of Biomedical Engineering, City University of Hong Kong, Tat Chee Avenue, Kowloon, Hong Kong, China

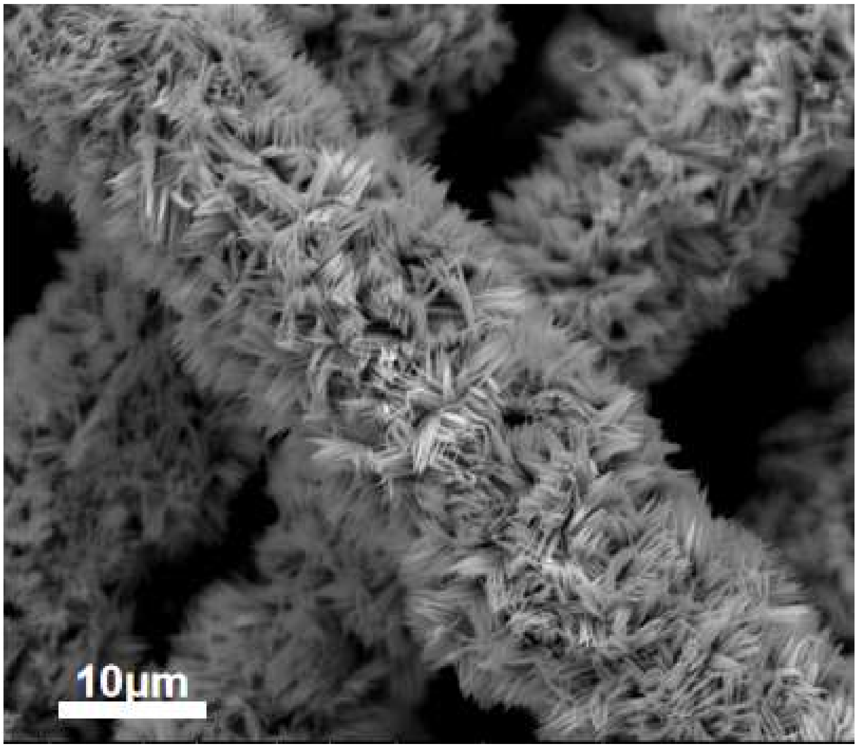

Figure S1. SEM image of $MnCo_2O_{4.5}$-Ar(600).

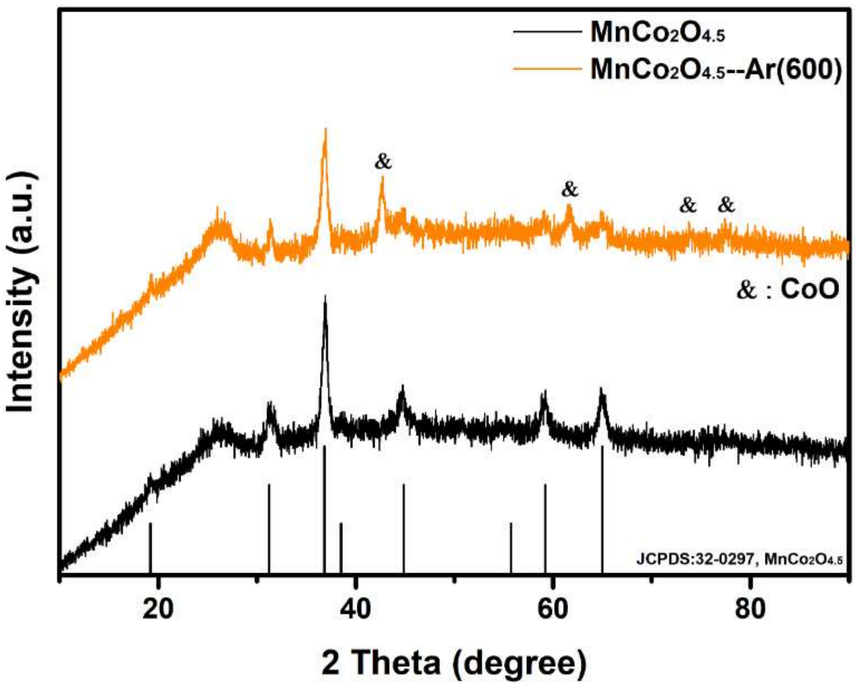

Figure S2. XRD patterns of the pristine $MnCo_2O_{4.5}$ and $MnCo_2O_{4.5}$-Ar(600).

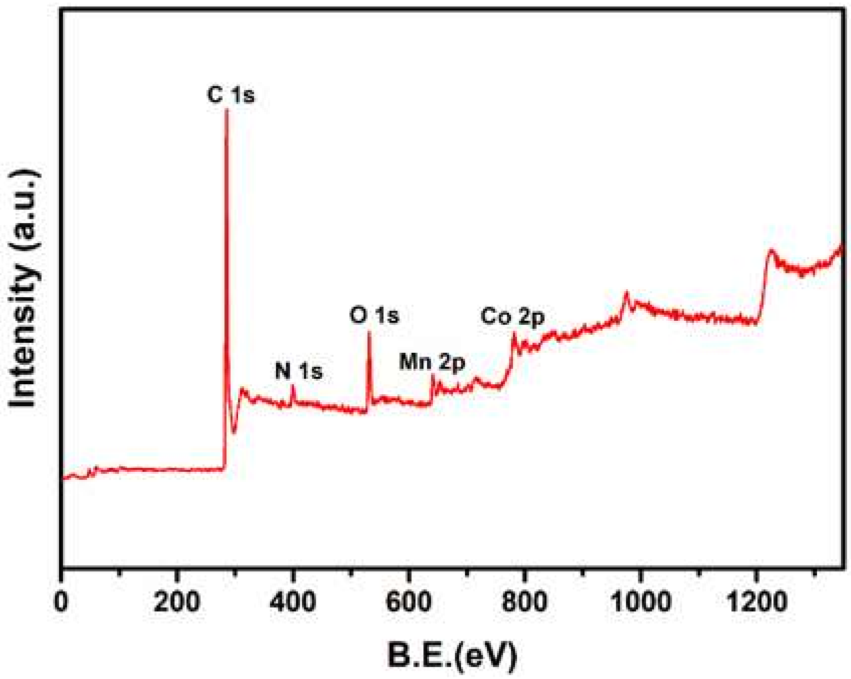

Figure S3. Survey scanning XPS spectra of the S-600.

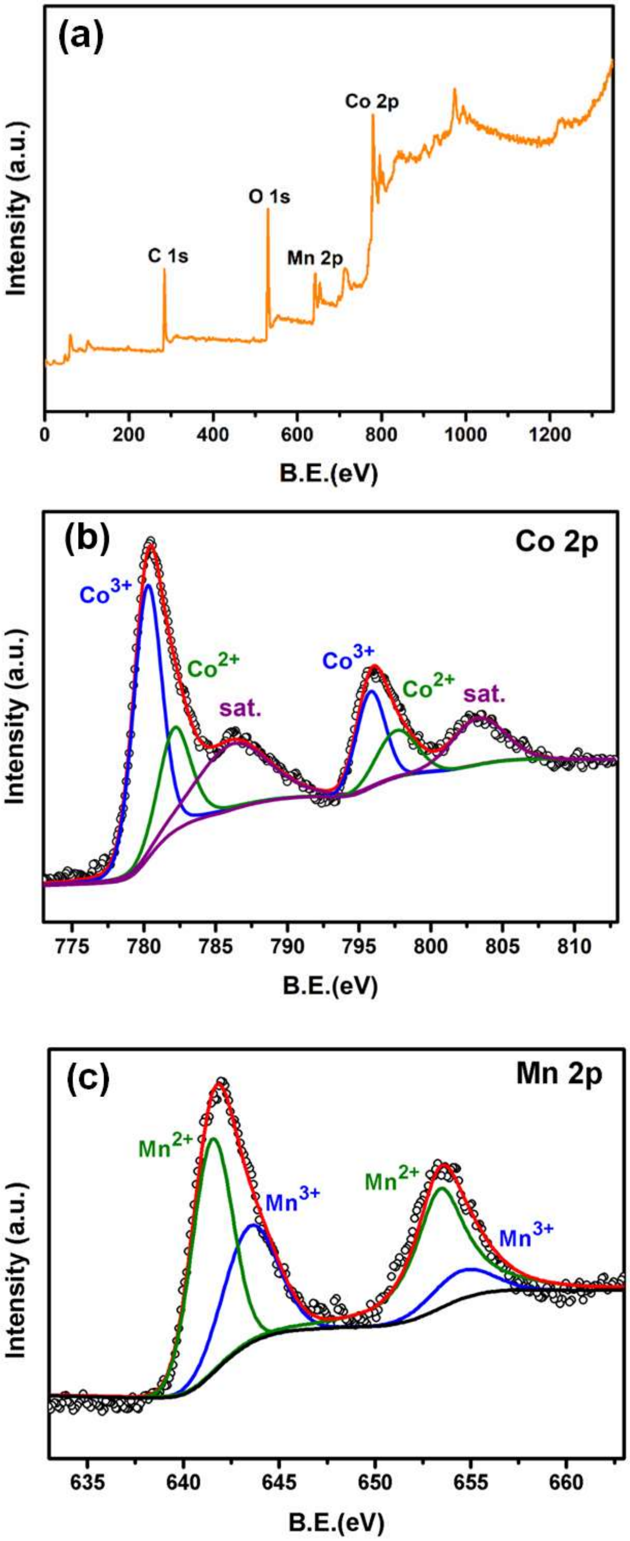

Figure S4. (a) Survey scanning XPS spectra of the $MnCo_2O_{4.5}$-Ar(600), (b)and (c) Corresponding high resolution Co 2p and Mn 2p.

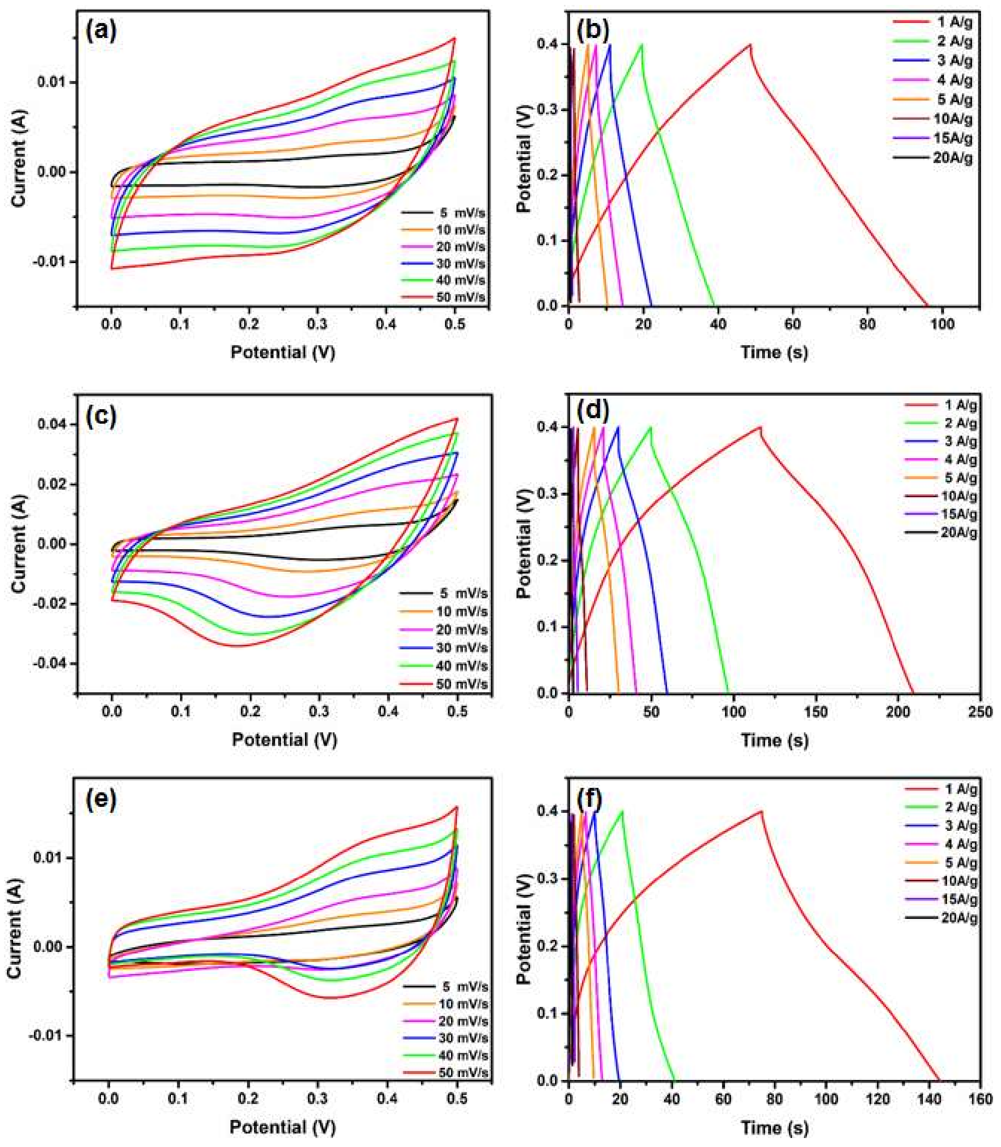

Figure S5. CV and GCD curves of (a-b) pristine $MnCo_2O_{4.5}$ NWs, (c-d) S-500 and (e-f) S-700.

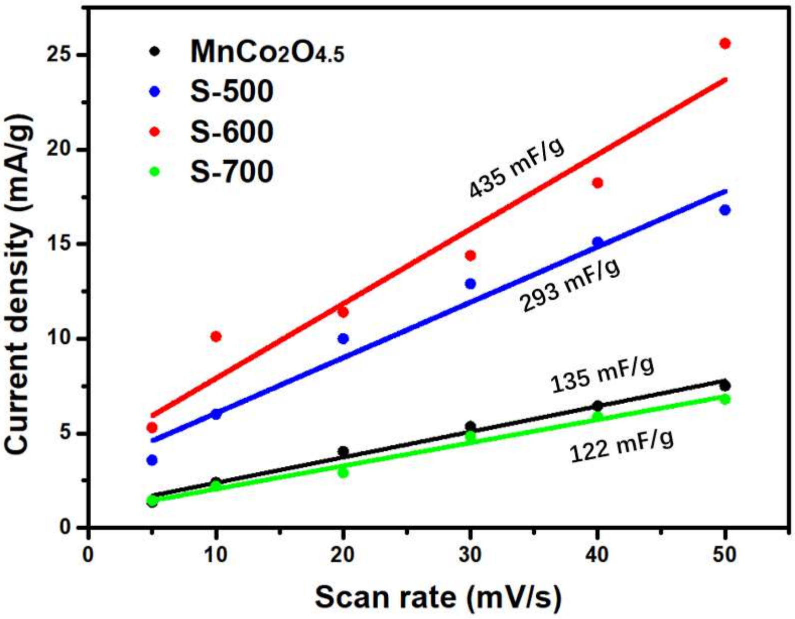

Figure S6. Current densities obtained from CV curves as a function of scanning rates, the corresponding slope being the C_{dl} values.

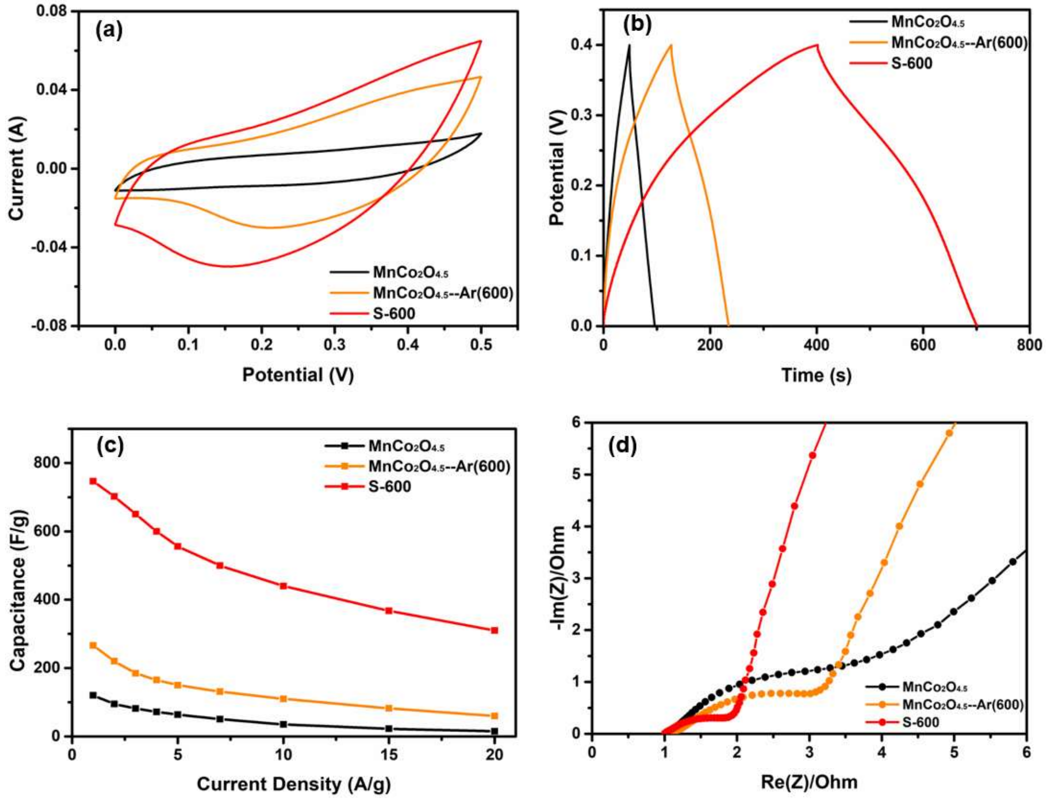

Figure S7. Electrochemical performance:(a) CV curves of $MnCo_2O_{4.5}$, $MnCo_2O_{4.5}$-Ar(600) and S-600 at a scanning rate of 50 mV s^{-1}; (b) GCD curves at 1 A g^{-1}; (c) Specific capacitances;(d) Nyquist plots

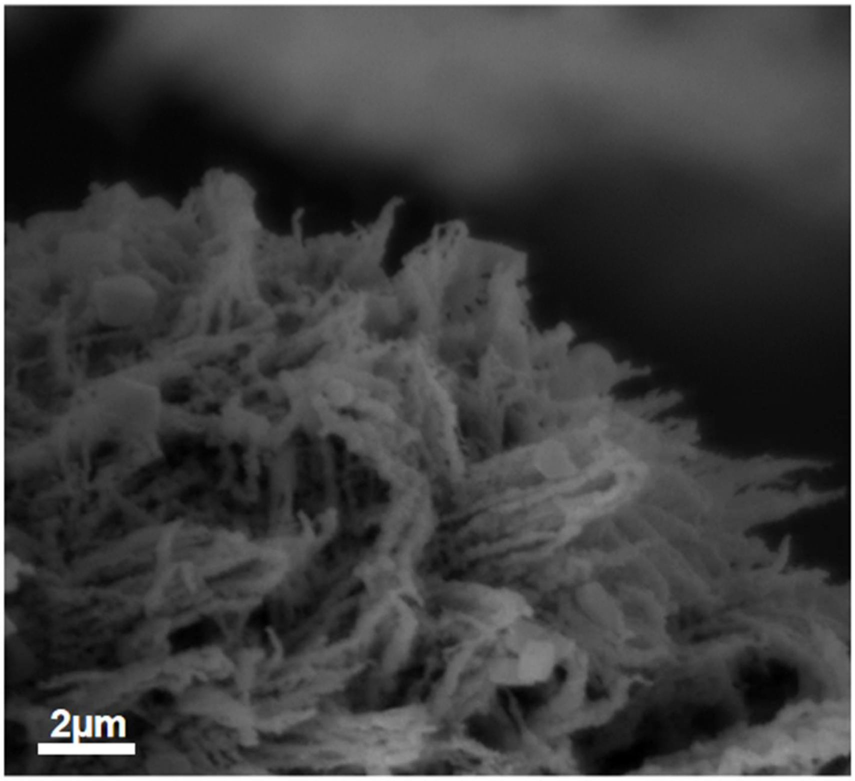

Figure S8. SEM image of S-600 NWs after 5000 cycles.

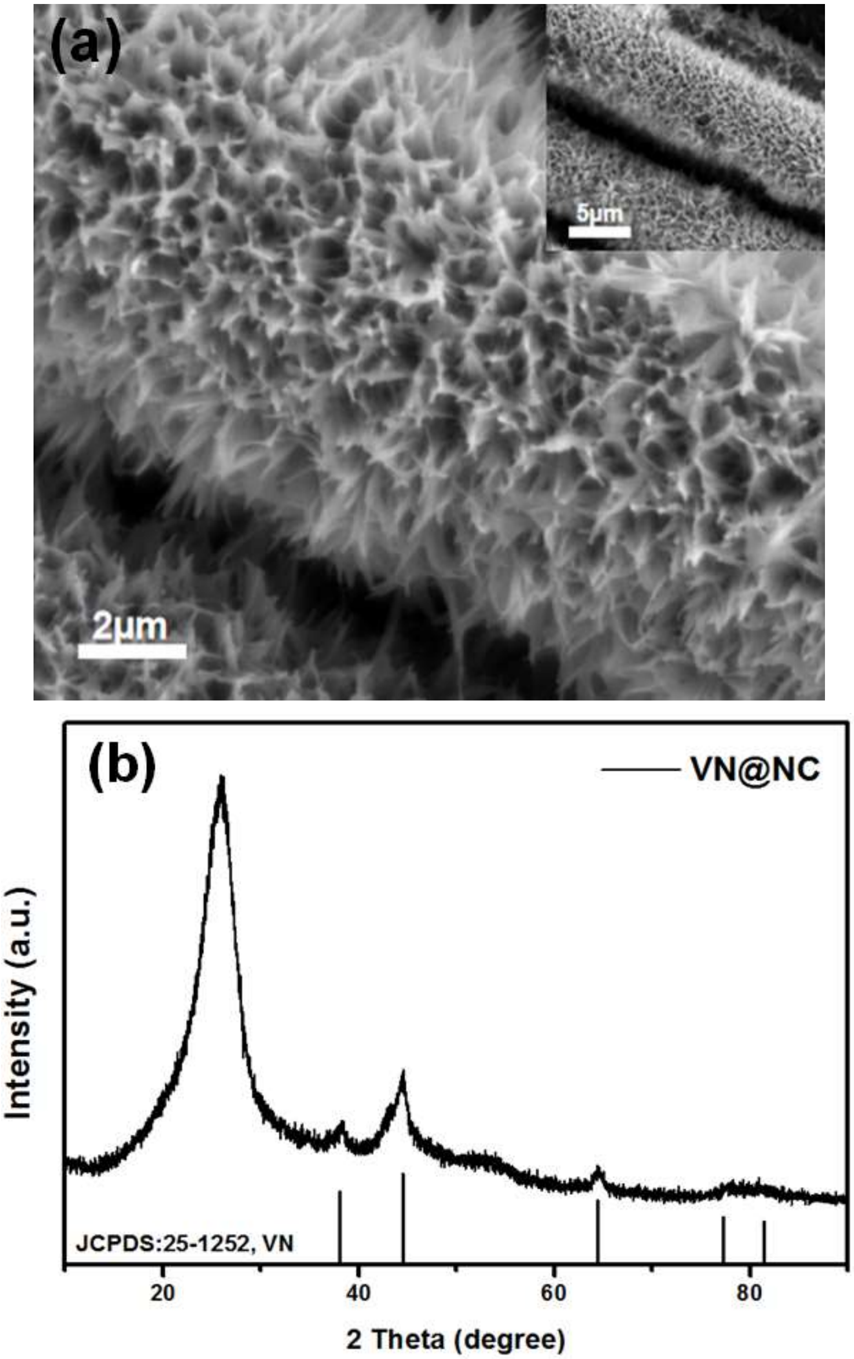

Figure S9. (a) SEM image of VN@NC; (b) Corresponding XRD pattern.

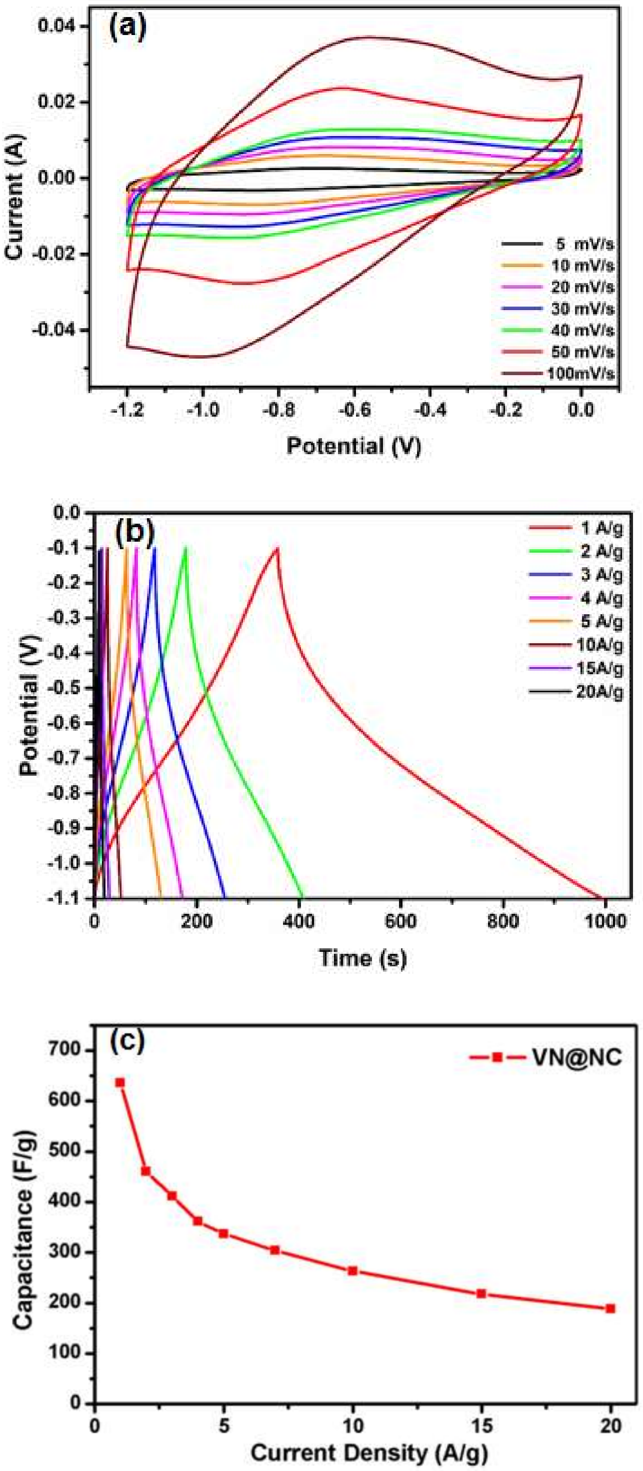

Figure S10. Electrochemical performance of VN@NC NWs electrode, (a) CV curves, (b) GCD curves, (c) Corresponding specific capacitances as function of discharging density.
